# Residual Hearing in DFNB1 Deafness and Its Clinical Implication in a Korean Population

**DOI:** 10.1371/journal.pone.0125416

**Published:** 2015-06-10

**Authors:** So Young Kim, Ah Reum Kim, Kyu Hee Han, Min Young Kim, Eun-Hee Jeon, Ja-Won Koo, Seung Ha Oh, Byung Yoon Choi

**Affiliations:** 1 Department of Otorhinolaryngology-Head and Neck Surgery, Seoul National University Hospital, Seoul National University College of Medicine, Seoul, Korea; 2 Department of Otorhinolaryngology-Head and Neck Surgery, National Medical Center, Seoul, Korea; 3 Department of Otorhinolaryngology-Head and Neck Surgery, Seoul National University Bundang Hospital, Seoul National University College of Medicine, Seongnam, Korea; 4 Sensory Organ Research Institute, Seoul National University Medical Research Center, Seoul, Korea; Universitat Pompeu Fabra, SPAIN

## Abstract

**Introduction:**

The contribution of Gap junction beta-2 protein (*GJB2*) to the genetic load of deafness and its mutation spectra vary among different ethnic groups.

**Objective:**

In this study, the mutation spectrum and audiologic features of patients with *GJB2* mutations were evaluated with a specific focus on residual hearing.

**Methods:**

An initial cohort of 588 subjects from 304 families with varying degrees of hearing loss were collected at the otolaryngology clinics of Seoul National University Hospital and Seoul National University Bundang Hospital from September 2010 through January 2014. *GJB2* sequencing was carried out for 130 probands with sporadic or autosomal recessive non syndromic hearing loss. The audiograms were evaluated in the *GJB2* mutants.

**Results:**

Of the 130 subjects, 22 (16.9%) were found to carry at least one mutant allele of *GJB2*. The c.235delC mutation was shown to have the most common allele frequency (39.0%) among *GJB2* mutations, followed by p.R143W (26.8%) and p.V37I (9.8%). Among those probands without the p.V37I allele in a *trans* configuration who showed some degree of residual hearing, the mean air conduction thresholds at 250 and 500 Hz were 57 dB HL and 77.8 dB HL, respectively. The c.235delC mutation showed a particularly wide spectrum of hearing loss, from mild to profound and significantly better hearing thresholds at 250 Hz and 2k Hz than in the non-p.V37I and non-235delC nonsyndromic hearing loss and deafness 1(DFNB1) subjects.

**Conclusion:**

Despite its reputation as the cause of severe to profound deafness, c.235delC, the most frequent DFNB1 mutation in our cohort, caused a wide range of hearing loss with some residual hearing in low frequencies. This finding can be of paramount help for prediction of low frequency hearing thresholds in very young DFNB1 patients and highlights the importance of soft surgery for cochlear implantation in these patients.

## Introduction

The incidence of profound pre-lingual hearing loss is about 1 per 1000 at birth and 1 per 300 children by 4 years of age [1]. Approximately 80% of all cases of genetic deafness, including all degrees of hearing loss, are non-syndromic, and in this group 60–75% exhibit autosomal recessive forms [2].

Mutations in the Gap junction β2 gene (*GJB2*) (OMIM 121011; DFNB1), which encodes connexin 26, are responsible for nearly half of the cases of hearing loss in many populations [3]. More than 100 mutations have been identified in *GJB2*, and most *GJB2* mutations are associated with recessive hearing loss [4].

The contribution of *GJB2* to the genetic load of deafness and its mutation spectra vary from mild to profound [5]. The audiograms associated with DFNB1 deafness are usually nonspecific [6].

Most *GJB2* mutations, including c.235delC and p.R143W, are associated with severe to profound hearing loss; however, some non-truncating mutations, including M34T, V37I, and L90P, are associated with a milder audiologic phenotype [5, 7–9]. In addition, a number of studies have addressed the progression of hearing loss associated with *GJB2* mutations [10–13]. The onset of DFNB1 hearing loss is usually congenital, but some cases demonstrate incomplete penetrance or delayed onset [7, 14–16].

The importance of residual hearing, especially at low frequencies, can critically affect the choice of electrodes and the electrode insertion depth during cochlear implantation for severe to profound hearing loss [17]. This is especially important in assessing infants, in whom measurement of the low-frequency hearing threshold is not always feasible. There have been rigorous studies dealing with audiologic features related to certain *GJB2* mutations in Caucasian populations [18–20] and East Asian populations [21]. Tsukada et al. (2010) and Snoeckx et al. (2005) reported that a non-truncating mutation, p.R143W, produced a more severe phenotype than other *GJB2* mutations [5, 21]. Nevertheless, there is still a paucity of studies addressing the frequency-specific residual hearing especially at low frequencies according to the type of *GJB2* mutations in East Asians. Therefore, we aimed to elucidate, if any, the correlation between certain kinds of *GJB2* mutations and the hearing thresholds at specific frequencies. In this study, we report that the most frequent *GJB2* mutation in East Asians, c.235delC, was associated with better residual hearing, especially at low frequencies [22].

## Materials and Methods

### Ethical considerations

The Institutional Review Boards (IRBs) of Seoul National University Bundang Hospital (SNUBH; Seongnam, South Korea) (IRB-B-1007-105-402) and Seoul National University Hospital (SNUH; Seoul, South Korea) (IRBY-H-0905-041-281) approved the study. We obtained written informed consent from all participants. For child participants, written informed consent was obtained from their parents or guardians.

### Study participants

An initial cohort of 588 subjects from 304 families with varying degrees of sensorineural hearing loss (SNHL) were collected at the otolaryngology clinics of SNUH and SNUBH from September 2010 through January 2014. Families segregating hearing loss apparently in an autosomal dominant, maternally-transmitted, or X-linked manner were excluded. Individuals with syndromic, unilateral hearing loss or with inner ear anomalies such as an enlarged vestibular aqueduct or incomplete partition type III were also excluded from this study. There was no age criterion for inclusion of subjects in this study. Resultantly, one-hundred and thirty subjects were selected based on the presence of autosomal recessive inheritance or sporadic occurrence of hearing loss without syndromic features. Whole blood (10 mL) was obtained from all 130 subjects. *GJB2* sequencing was performed for each family. Those subjects with *GJB2* mutations were then classified by the genotype (including a combination of two mutant alleles of *GJB2* in a *trans* configuration). Next, hearing thresholds were determined for each *GJB2* genotype.

### Clinical evaluation

The following demographic data were collected for each patient: sex, birth date, date of initial otolaryngological consultation, and major comorbidities or syndromic features. The ethnicity of all subjects was Korean. Where available, temporal bone computed tomography data or magnetic resonance imaging findings were recorded (e.g., anomalies in the eighth nerve, cochlear anomalies, or an enlarged vestibular aqueduct).

### Audiometric evaluation

All subjects underwent an audiological evaluation. Hearing levels were determined by pure-tone audiometry. We performed serial audiograms for average (SD) 3.1 (3.5) years to detect any noticeable aggravation of hearing after their participation in this study and to provide timely auditory rehabilitation. At each test frequency, if there was a noticeable change in hearing threshold, the poorest threshold was used for analysis. In addition, when necessary we traced back a subject’s previous hearing status by obtaining clinical information from the referring private practitioner. If there was noticeable change during the serially performed or retrospective reviewed audiogram, we employed the worst hearing threshold to analyze in this study. When at least one recessive mutant allele of *GJB2* was detected, we explored any residual hearing across all frequencies. In detail, the pure-tone thresholds at 0.25, 0.5, 1, 2, 4 and 8 kHz were all recorded with exceptions of some infants where pure-tone thresholds from only limited frequencies could be obtained due to limited reliable responses. Residual hearing was defined as a hearing threshold in pure tone audiometry <80 dB at any specific frequency. The hearing level of the better ear, calculated from the four-tone average (0.5, 1, 2, and 4 kHz), was labeled as subtle (16–25 dB), mild (26–40 dB), moderate (41–70 dB), severe (71–95 dB), or profound (>95 dB).

### Polymerase chain reaction (PCR) amplification and Sanger sequencing of *GJB2*

As a first step toward making a molecular genetic diagnosis for the 130 subjects, we performed a bidirectional nucleotide sequence analysis of the two exons of *GJB2* (GenBank Accession NM_004004.5). The two exons and flanking sequences of *GJB2* were PCR-amplified and sequenced as described previously [23]. The mutation nomenclature and nucleotide numbering were based on NM_004004.5 (*GJB2* cDNA). The names of all variants were checked using Mutalyzer [24].

### Statistical analysis

The SPSS18.0 statistical package was used in our analysis (IBM, Armonk, NY, USA). The Levene’s test was conducted to compare the variance between non-p.V37I groups with and without c.235delC. The Mann-Whitney U test was used to compare the mean audiologic threshold at each frequency according to *GJB2* genotypes.

## Results

Of 130 unrelated hearing-impaired mostly pediatric subjects who did not exhibit any inner ear abnormality and therefore underwent *GJB2* sequencing for candidacy of DFNB1, 22 subjects (16.9%) were found to carry at least one mutant allele of *GJB2*. A total of ten different mutations and thirteen different genotypes (combination of two mutant alleles of *GJB2* in a *trans* configuration) were identified in our cohort (Table 1). The overall frequency of the *GJB2* mutant allele among the 260 alleles from 130 hearing impaired subjects was 15.8% (41/260) (Table 2). The most common *GJB2* mutation in the population was c.235delC, of which the allele frequency was 39.0% (16/41) among the 41 detectable mutant alleles of *GJB2* in our cohort, followed by p.R143W (11/41 [26.8%]) and p.V37I (4/41 [9.8%]) (Table 1). These three dominant mutations, c.235delC, p.R143W, and p.V37I, accounted for 75.6% of the total *GJB2* mutation alleles detected in our cohort (Table 1).

**Table 1:** *GJB2* mutations and their allele frequencies.

GJB2 mutation	Homozygote	Compound heterozygote	Single heterozygote	Number of mutation alleles	Allele frequency in GJB2 candidates (in 260 allele) (%)	Allele frequency in GJB2 mutants (in 41 allele) (%)
**c.235delC**	2	11	1	16	6.2	39.0
**p.R143W**	1	8	1	11	4.2	26.8
**p.V37I**	0	3	1	4	1.5	9.8
**c.299_300delAT**	0	3	0	3	1.2	7.3
**c.176_191del**	0	2	0	2	0.8	4.9
**p.Glu47X(c.139G>T)**	0	1	0	1	0.4	2.4
**c.508_511dupAACG**	0	1	0	1	0.4	2.4
**p.T86R**	0	1	0	1	0.4	2.4
**p.Asp159Val**	0	1	0	1	0.4	2.4
**p.Val193Glu**	0	1	0	1	0.4	2.4
**Total**	3	16	3	41	15.8	100.0

**Table 2:** *GJB2* mutants and their hearing levels in this study.

*GJB2* mutations	No. of subjects	Mean age at recruitment(age)	Average onset of hearing loss (age)	Average thresholds* (dB)	Low frequency average† (dB HL)
**p.V37I/p.R143W**	2	11±5.7	6.3±1.8	60.8±13.0	59.2±13.0
**p.V37I /c.235delC**	1	15	11	25	23.3
**p.V37I single hetero**	1	3	3	65	55
**p.R143W homozygote**	1	1	1	112.5	108.3
**p.R143W/c.235delC**	5	0.9±0.5	0.9±0.5	93.5±22.7	90.7±26.8
**p.R143W/p.T86R**	1	0.9	0.9	110	75
**p.R143W single heterozygote**	1	3	3	92.5	80
**c.235delC homozygote**	2	4.3±2.5	2±1.4	74.2±57.8	69.2±64.8
**c.235delC compound heterozygote(3 with c.299_300delAT, 2 with c.176_191del)**	5	1.5±0.5	1±0.6	88.5±14.5	85±24.5
**c.235delC single heterozygote**	1	2	2	82.5	110
**p.Glu47X(c.139G>T)/c.508_511dupAACG**	1	0.9	0.9	110	100
**p.Asp159Val/p.Val193Glu**	1	0.8	0.8	117.5	105
**Total**	22	3.1±3.5	2.2±3.9	87.2±26.3	82.3±27.6

(*average of 500, 1000, and 2000 Hz)

(†average of 250, 500, 1000 Hz)

The *GJB2* mutation (+) group (n = 22) consisted of 14 males (63.6%) and 8 females (36.4%) with ages ranging from 9 months to 25 years; 95.5% (21/22) of the subjects were within the age range of 0–15 years. The mean age at recruitment to the study was 3.1 (±3.5) years. Among these 22 subjects, 19 subjects were confirmed to be DFNB1, carrying two mutant alleles of *GJB2* either as a homozygous or a compound heterozygous state (Table 1). There were three single heterozygotes (c.235delC (SH107-225), p.R143W (SH60-136) and p.V37I (SB80-141)) (Table 1). To better address whether these subjects are truly DFNB1 or not, we conducted a multiplex breakpoint PCR assay to detect, if any, the reported large genomic deletions that might reside in the DFNB1 locus in *trans* with the detected *GJB2* mutation [18, 24] from two subjects (SH107-225 and SB80-141) and performed targeted exome sequencing (TES) from one subject (SH60-136). We did not detect any genomic deletion in the DFNB1 locus from SH107-225 and SB80-141. However, we reason that SB80-141 carrying p.V37I is likely to be DFNB1, based upon degree of hearing loss of SB80-141 as previously suggested [23]. The same reasoning was used to attribute the severe SNHL of SH107-225 to p.235delC. This is the most prevalent GJB2 mutation in East Asians [25]. SH60-136 turned out not to carry any other convincing variant in the 80 reported deafness genes [26], making SH60-136 likely to be DFNB1.

**Fig 1:**
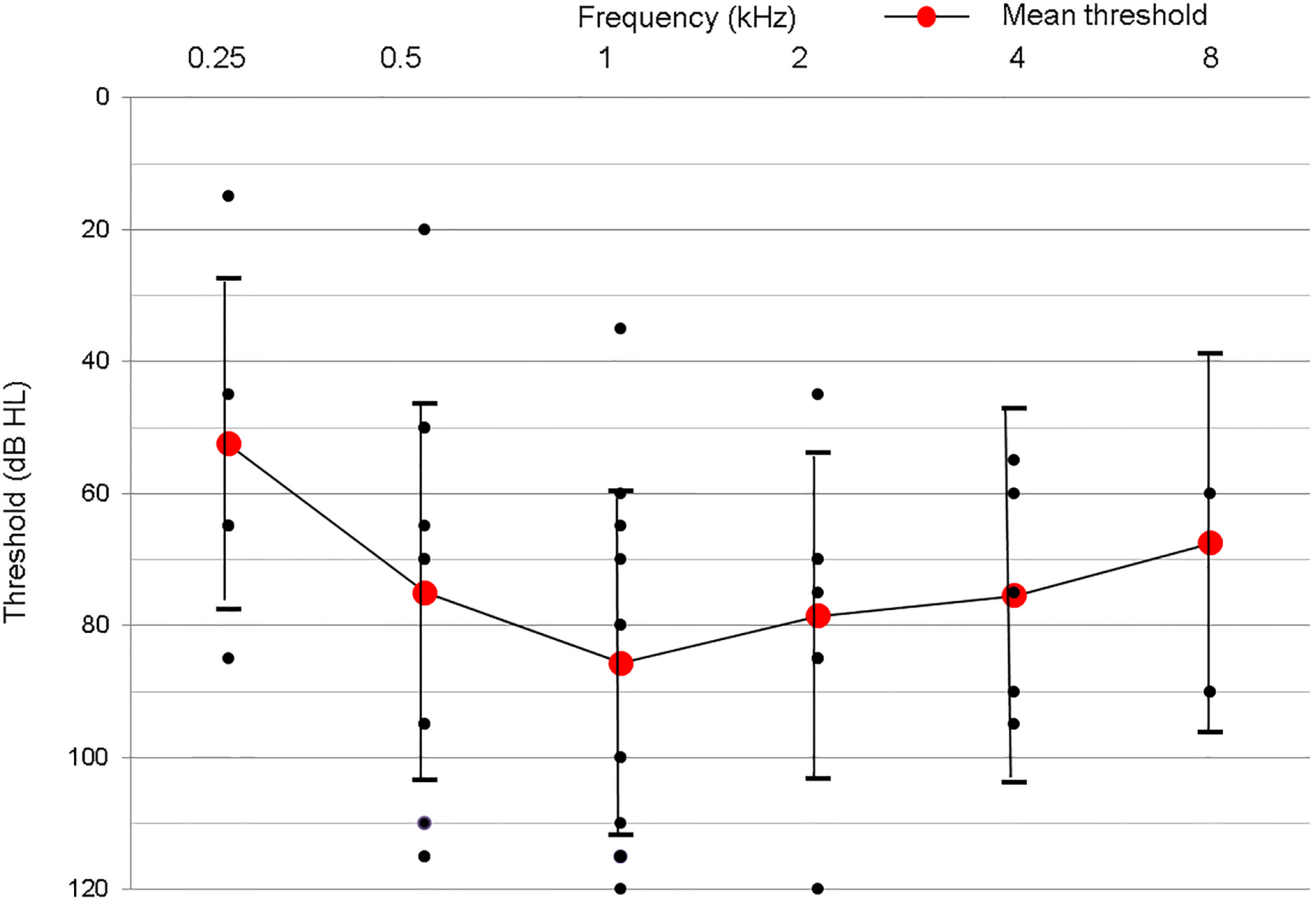
Overlapping audiograms of all patients with *GJB2* mutations. All of the patients with hearing loss caused by *GJB2* mutations in this study (n = 22). (Black dots mean hearing thresholds of each subject, which may represent more than one subject with identical hearing thresholds. Red dots represent mean hearing thresholds of each frequency).

**Fig 2:**
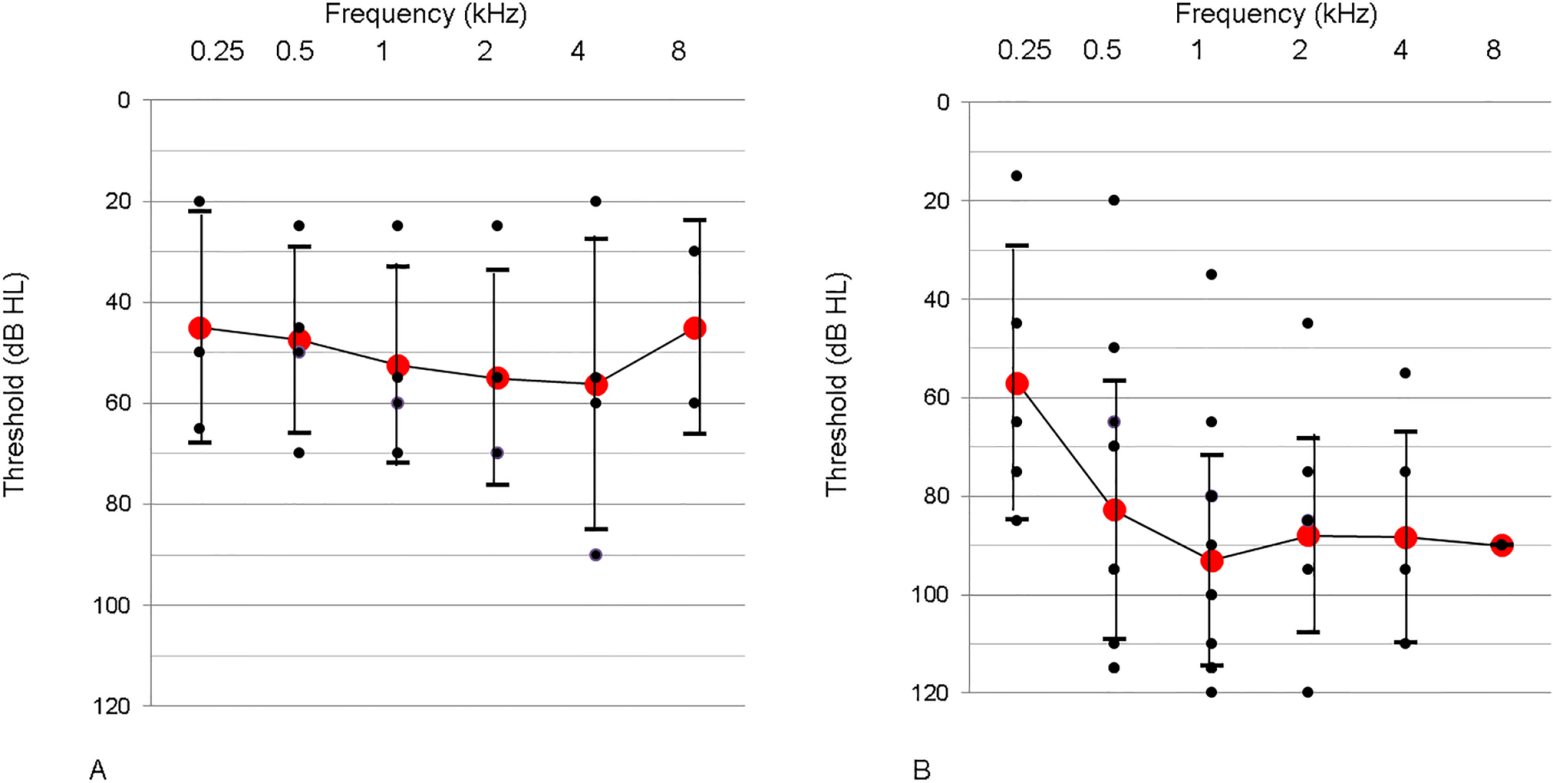
Overlapping audiograms of the patients with p.V37I and non-p.V37I *GJB2* mutations. A. Patients with hearing loss caused by a p.V37I mutation (n = 4). B. Patients with hearing loss caused by a non-p.V37I mutation (excluding patients with a p.V37I mutation in the *trans* configuration) (n = 18). (Black dots mean hearing thresholds of each subject, which may represent more than one subject with identical hearing thresholds. Red dots represent mean hearing thresholds of each frequency).

These DFNB1 subjects including the three single heterozygotes in our cohort manifested various levels of SNHL (Fig 1). Table 2 shows the distribution of average hearing thresholds according to genotype. p.V37I was identified in four subjects, three of whom were identified as carrying one of two mutant alleles of *GJB2*. Those DFNB1 subjects carrying p.V37I (n = 4) showed clearly better hearing thresholds than the other DFNB1 subjects (Table 2 and Fig 2) (p = 0.01, Mann-Whitney U test). One of the subjects (SB42-94) carrying c.235delC in a *trans* configuration with p.V37I showed the best threshold (mean threshold: 24.2 dB), as compared to the other subjects with p.V37I (Table 2). The p.V37I subjects had a significantly older mean onset age of hearing loss (6.7 years), as compared to the non-p.V37I subjects (1.2 years) (p<0.001, Mann-Whitney test).

**Fig 3:**
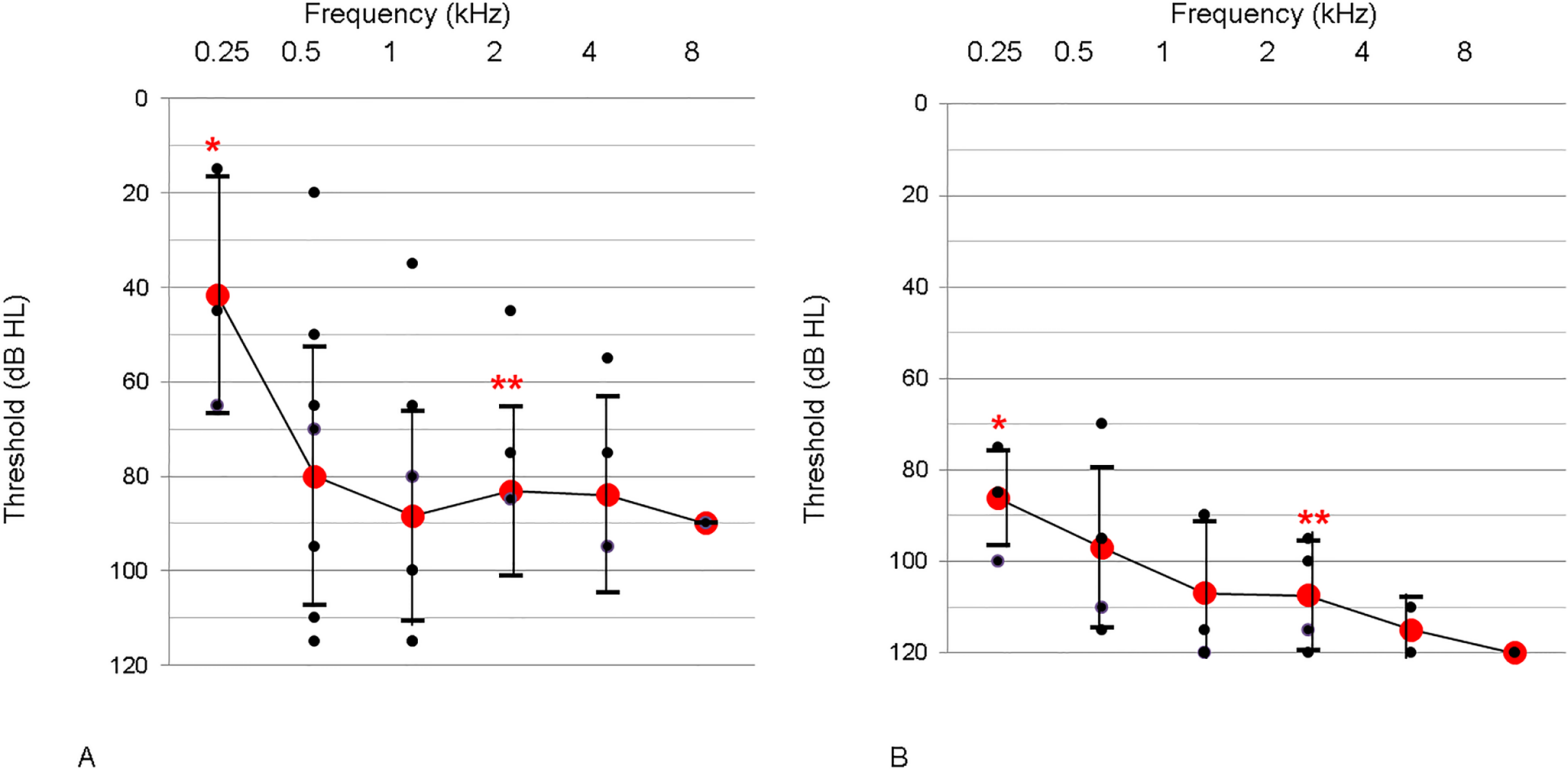
Overlapping audiograms of the patients with c.235delC and non-c.235delC *GJB2* mutations. A. Patients with hearing loss caused by a c.235delC mutation (excluding patients with a p.V37I mutation in the *trans* configuration) (n = 13). B. Patients with hearing loss caused by a non-c.235delC mutation (excluding patients with a p.V37I mutation in the *trans* configuration) (n = 5). Detailed lists of non-c.235delC mutations are p.R143W homozygote, p.R143W single heterozygote, p.Glu47* and p.Ala171Glufs*40 compound heterozygote, and p.T86R and p.R143W compound heterozygote. (*p = 0.05, **p = 0.03, by the Mann-Whitney U test) (Black dots mean hearing thresholds of each subject, which may represent more than one subject with identical hearing thresholds. Red dots represent mean hearing thresholds of each frequency).

**Fig 4:**
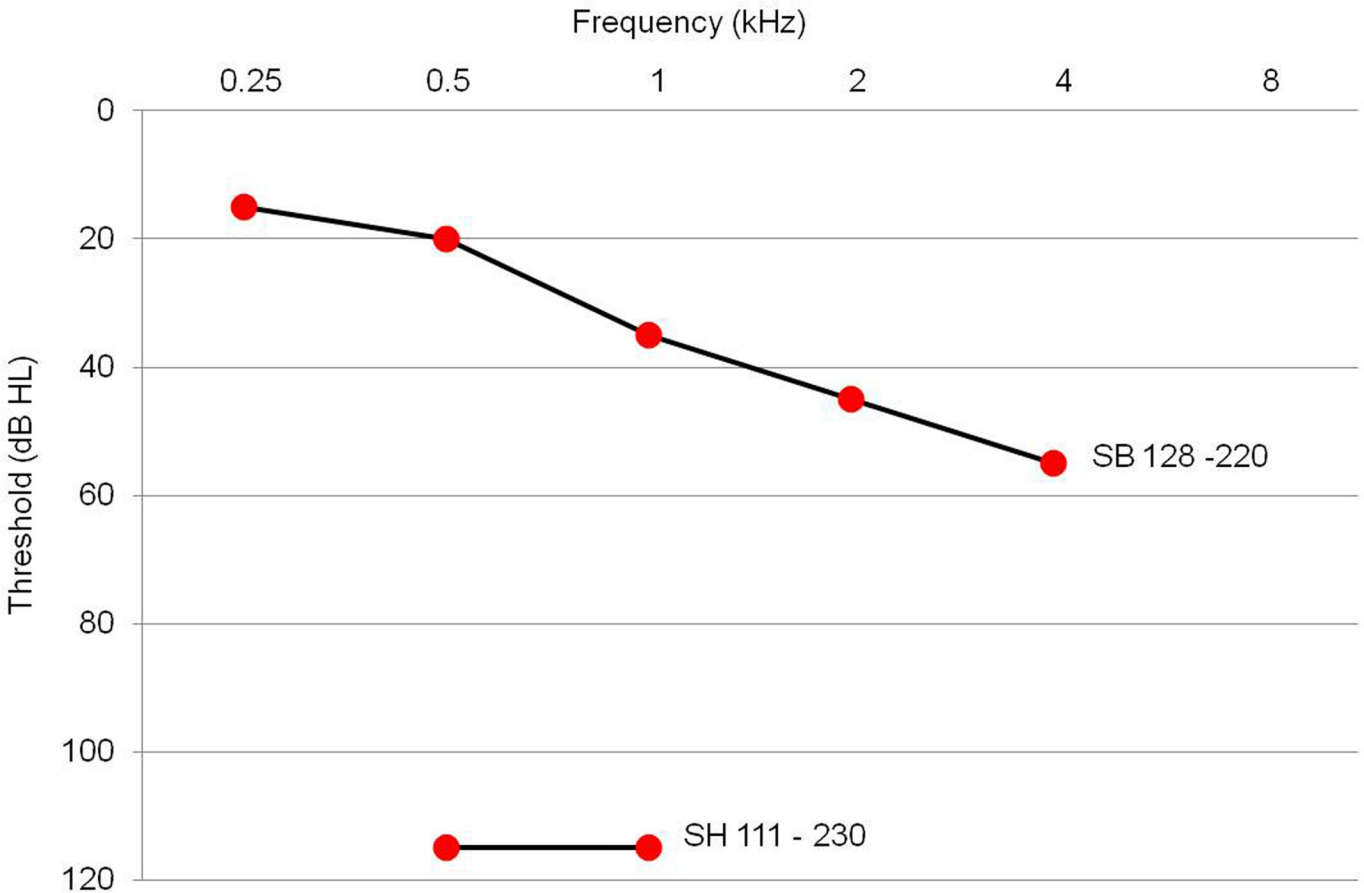
Overlapping audiograms from two subjects who were c.235delC homozygotes. The subjects showed dramatically different levels of hearing loss, with one subject showing moderate hearing loss (mean hearing threshold, 38.8 dB) and the other showing profound hearing loss (mean hearing threshold, 110 dB).

The non-p.V37I DFNB1 subjects (n = 18) exhibited a mean hearing threshold of 93.5 dB (Fig 2), again confirming *GJB2* mutations as an important cause of pre-lingual severe to profound SNHL in this Korean cohort. Notably, they retained some degree of residual hearing, leading to hearing thresholds of 57 dB HL and 82.9 dB HL at 250 and 500 Hz, respectively. This audiogram configuration is different from the relatively flat audiogram over all frequencies for the p.V37I DFNB1 subjects (Fig 2). Breaking down the hearing results according to genotype, the most frequent *GJB2* mutation in the non-p.V37I DFNB1 subjects, c.235delC, caught our attention due to the wide range of hearing thresholds and relatively good residual hearing—especially at low frequencies (Fig 3). Although the variance between the non-p.V37I groups with and without c.235delC was not significantly different due to the small sample number, the non-p.V37I group with c.235delC subjects demonstrated a wide range of hearing thresholds. Notably, two c.235delC homozygotes (SH 111–230 and SB 128–220) showed dramatically different levels of hearing loss (Fig 4). When we compared the hearing thresholds of all measured frequencies and width of the hearing threshold ranges of the non-p.V37I DFNB1 subjects carrying c.235delC with those of non-p.V37I and non-c.235delC DFNB1 subjects, those DFNB1 subjects who carried c.235delC showed a significantly better hearing threshold than did the non-p.V37I and non-c.235delC DFNB1 subjects at 250 Hz and 2 kHz, respectively (p = 0.05, 0.03, Mann-Whitney U test) (Fig 3).

## Discussion

Mutations in *GJB2* are known to be responsible (though not always) for non-syndromic SNHL, which is commonly related with a severe to profound degree of autosomal recessive inheritance [5, 6]. However, some non-truncating mutations, which may have a lesser deleterious effect on cochlear function and hearing (e.g., p.V37I and p.M34T), generally produce mild to moderate hearing loss and a later onset of hearing loss [5, 7, 9]. Our study confirmed that p.V37I-related DFNB1 produced a milder auditory phenotype than non-p.V37I DFNB1, as described previously in this population [23].

The c.235delC mutation, the most frequent *GJB2* mutation in East Asian populations, was previously shown to cause a severe to profound auditory phenotype [27–29]. It was proposed that truncating mutations (e.g., c.35delG and c.235delC) caused more severe hearing loss than non-truncating mutations (e.g., p.V37I and L90P) [18, 28]. Specifically, the c.35delC, well-known truncating *GJB2* mutation in Caucasian, rarely showed residual hearing, except in some cases in a *trans* configuration with some mutations with a mild pathogenic potential such as c.292C>T(p.R98W) [30, 31]. However, our data show that c.235delC caused a wide spectrum of hearing loss (from mild to profound) in this population even in the homozygote (SB128-220) (Fig 4). Compared to subjects carrying the c.235delC mutation, DFNB1 subjects without c.235delC showed worse hearing thresholds. This observation could partly reflect the auditory phenotypes of p.R143W, which comprised the largest part of non-p.V37I, non-c.235delC DFNB1 in our cohort. The p.R143W mutation is a non-truncating missense mutation that causes the production of non-functional channels [32]. Although it is not a truncating mutation, and the functional mechanism is not clearly defined, the p.R143W mutant allele has been consistently associated with a severe auditory phenotype [5, 21]. In a previous study, subjects who were c.35delG/p.R143W showed more severe hearing loss than 35delG homozygotes [5]. Likewise, patients with c.235delC/p.R143W showed more severe hearing loss than those with other c.235delC-containing phenotypes [21]. In our study, we focused on the auditory phenotype of non-p.V37I DFNB1 subjects carrying at least one allele of c.235delC, revealing that these cases manifested significant residual hearing at 250 Hz and that their mean hearing thresholds fell within the range of severe SNHL at higher frequencies, rather than profound SNHL (Fig 3). This relatively less severe auditory phenotype related to c.235delC was again contrasted with hearing thresholds from very young subjects carrying Myosin XVA *(MYO15A)* mutations in our cohort (S1 Fig) [33].

Furthermore, two homozygotes for c.235delC showed a significantly different degree of SNHL (Fig 4). Such phenotypic variability from an identical genotype has been attributed to unknown modifier genes or environmental factors leading to incomplete penetrance and variable expressivity [34]. Several previous studies dealing with c.235delC focused on subjects with severe to profound SNHL [35, 36]. Some mildly hearing-impaired DFNB1 subjects carrying c.235delC would not have been included if the study population was restricted to only severe to profound SNHL. To avoid this selection bias, our unique cohort included subjects with various degree of SNHL and this broad inclusion criterion about the degree of hearing loss helped to clearly identify or confirm certain *GJB2* mutant alleles associated with the better residual hearing.

The presence of residual hearing can affect the planning and placement of cochlear implants. Recent studies demonstrated that children with *GJB2*-related hearing loss showed a better auditory outcome after cochlear implantation than age-matched children [37]. This was explained in previous studies by a normal spiral ganglion cell count [38] or an isolated insult to the cochlea that allowed for the preservation of central cognitive function in DFNB1 hearing loss [39, 40]. Our study suggests that the substantial exposure to the sound preoperatively relying on the presence of residual hearing can also contributes to the reported better outcome for some DFNB1 subjects. Our result proposed that knowing the genotype of *GJB2* can potentially provide us the clue to the audiologic phenotype, especially at low frequencies which is sometimes not feasible to evaluate in infants.

## Conclusion

This study showed wide range of hearing loss and low frequencies residual hearing in the DFNB1 subjects with the c.235delC mutation. Especially for patients with residual hearing, it is important to try to preserve this residual hearing during cochlear implantation. Clinicians should keep in mind that there may be substantial residual hearing, especially at low frequencies, in DFNB1 subjects related to c.235delC in Koreans. A further study with a larger cohort and more longitudinal data will improve our knowledge on the residual hearing associated with various *GJB2* mutations. This can be of importance when planning the insertion depth of cochlear implants in infants, in whom the measurement of low-frequency hearing thresholds may not be feasible.

## Supporting Information

S1 Fig Overlapping audiograms from three subjects with *MYO15A* mutations. All three patients showed profound hearing loss and, rarely, residual hearing.
(TIF)
