# Supplementary figures and images for: Residual Hearing in DFNB1 Deafness and Its Clinical Implication in a Korean Population
Source: PLoS One. 2015 Jun 10;10(6):e0125416. doi: 10.1371/journal.pone.0125416 (PMC4464755; doi:10.1371/journal.pone.0125416)

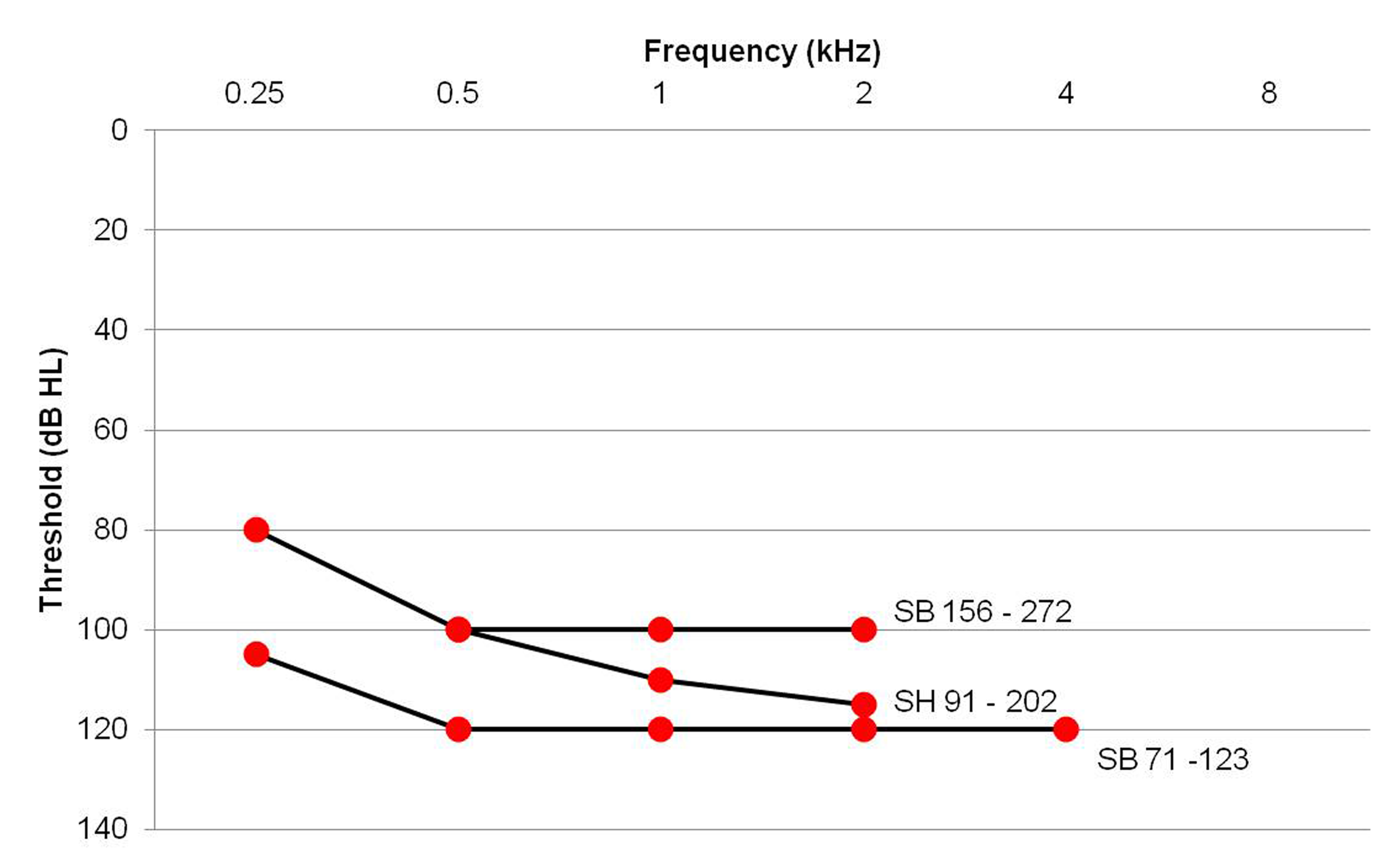

Supplement: S1 Fig — All three patients showed profound hearing loss and, rarely, residual hearing. (TIF) [file pone.0125416.s001.tif]
